# circAGFG1 sponges miR-28-5p to promote non-small-cell lung cancer progression through modulating HIF-1α level

**DOI:** 10.1515/med-2021-0269

**Published:** 2021-05-05

**Authors:** Xiaoan Ma, Cuijie Wang, Juan Chen, Dan Wei, Fei Yu, Juan Sun

**Affiliations:** Department of Respiratory Medicine, No. 215 Hospital of Shaanxi Nuclear Industry, Xianyang 712000, Shannxi, China

**Keywords:** NSCLC, circAGFG1, miR-28-5p, HIF-1α, glycolysis

## Abstract

Circular RNAs (circRNAs) have gained much attention for their crucial regulatory roles in human diseases and cancers. However, the role and the mechanism of circRNA ArfGAP with FG repeats 1 (circAGFG1) in non-small-cell lung cancer (NSCLC) are still largely unknown. circAGFG1 was highly expressed in NSCLC, and high expression of circAGFG1 was closely related to the low survival rate of NSCLC patients. circAGFG1 knockdown inhibited the proliferation, migration, and invasion and promoted the apoptosis of NSCLC cells. circAGFG1 bound to miR-28-5p in NSCLC cells, and circAGFG1 promoted NSCLC progression partly through sponging miR-28-5p *in vitro*. HIF-1α was a target of miR-28-5p, and miR-28-5p overexpression-mediated influences in NSCLC cells were partly overturned by the addition of HIF-1α overexpression plasmid. circAGFG1/miR-28-5p/HIF-1α axis regulated cellular glycolytic metabolism in NSCLC cells. circAGFG1 silencing restrained the xenograft tumor growth *in vivo*. circAGFG1 promoted the proliferation, migration, and invasion and suppressed the apoptosis of NSCLC cells through accelerating the glycolysis via miR-28-5p/HIF-1α axis.

## 1 Introduction

Non-small-cell lung cancer (NSCLC) is a common cancer and results in a great burden on the society. Therefore, it is meaningful to screen effective molecular targets for NSCLC. An increasing number of circular RNAs (circRNAs) has been identified in recent years [1]. circRNAs have been found as important modulators in many biological behaviors [2]. Accumulating articles have pointed out the regulatory functions of circRNAs in the progression of NSCLC [3,4]. circRNA ArfGAP with FG repeats 1 (circAGFG1) played an oncogenic function in many malignancies, including cervical cancer [5], triple-negative breast cancer [6], and NSCLC [7]. Nevertheless, the biological function of circAGFG1 in the development of NSCLC remains to be disclosed.

MicroRNAs (miRNAs) could function as posttranscriptional modulators of messenger RNAs (mRNAs) to degrade mRNAs or suppress the translational process [8,9]. miRNAs are also reported to regulate the initiation and the progression of diverse malignancies, including NSCLC. For instance, miR-125a restrained cervical cancer progression [10]. miR-16-1-3p hampered the proliferation ability and motility in NSCLC cells via targeting TWIST1 [11]. miR-28-5p was reported to inhibit the development of pancreatic cancer [12] and colon cancer [13]. However, the role of miR-28-5p in NSCLC is still unclear.

Hypoxia-inducible factor 1 (HIF-1) acts as the transcription factor to exhibit a crucial role in cellular hypoxic adaptation. HIF-1α is a subunit of HIF-1 [14,15]. Targeting HIF-1α signaling is an important direction for the treatment of cancers, including gastric cancer and NSCLC [16,17]. Here, we found a novel signal axis of circAGFG1/miR-28-5p/HIF-1α that was implicated in the regulation of NSCLC development.

Cancer cells exhibit a unique metabolic phenotype, termed as the Warburg effect, featured by increased glycolytic metabolism and decreased oxidative phosphorylation, even with the presence of oxygen [18]. The Warburg effect provides growth advantages for cancer cells under the hypoxic tumor microenvironment, and it also decreased the release of reactive oxygen species in mitochondria [19,20]. Nevertheless, the potential mechanism behind the glycolysis of cancer cells remains to be disclosed.

circAGFG1 abundance was abnormally enhanced in the serum of NSCLC patients and NSCLC tissues compared with their corresponding controls. The role and the downstream signal network of circAGFG1 in NSCLC cells were explored.

## 2 Materials and methods

### 2.1 Sample collection

A total of 45 serum samples from patients who were diagnosed with NSCLC and 25 serum samples from healthy volunteers in No. 215 Hospital of Shaanxi Nuclear Industry were collected. Also, 45 pairs of tumor tissues and adjacent nontumor tissues from NSCLC patients were collected. All the participants had signed informed consent before surgery or serum sample collection. This study had obtained permission from Ethics Committee of No. 215 Hospital of Shaanxi Nuclear Industry.

### 2.2 Cell culture

Human bronchial epithelial cell line (HBE1) and a panel of four NSCLC cell lines (A549, 95-D, HCC827, and H1299) were obtained from BeNa Culture Collection (Beijing, China). All cell lines were maintained in Dulbecco’s modified Eagle’s medium (DMEM; Gibco, Carlsbad, CA, USA) added with 10% fetal bovine serum (FBS; Gibco) and penicillin/streptomycin (100 units/mL) at 37℃ in a 5% CO_2_ environment.

### 2.3 Real-time quantitative polymerase chain reaction

PrimeScript RT Reagent Kit (for circAGFG1, AGFG1, and HIF-1α; Takara, Dalian, China) and TaqMan MicroRNA Reverse Transcription kit (for miR-28-5p; Applied Biosystems, Rotkreuz, Switzerland) were used to obtain template DNA. PCR reaction was conducted with Real Master Mix (SYBR Green) (Tiangen, Beijing, China). Housekeeping genes, including *U6* (for miR-28-5p) and glyceraldehyde-3-phosphate dehydrogenase (GAPDH; for circAGFG1, AGFG1 and HIF-1α), were used as internal controls. The relative enrichment of circAGFG1, AGFG1, miR-28-5p, and HIF-1α was calculated by the 2^−ΔΔCt^ formula. Special primers were presented in Table 1.

**Table 1:** Primers in RT-qPCR

Gene	Species	Direction	Sequence (5′–3′)
circAGFG1	Human	Forward	CCAGTTGTAGGTCGTTCTCAAG
		Reverse	TCACCCTGTGTGGTGGAT
AGFG1	Human	Forward	AAGTGAAAGAGTTTCTACAA
		Reverse	GCAGTGTTGGTGCAGAATCC
miR-28-5p	Human	Forward	GGGAAGGAGCTCACAGTCT
		Reverse	CAGTGCAGGGTCCGAGGTAT
HIF-1α	Human	Forward	ACCTATGACCTGCTTGGTGC
		Reverse	GGCTGTGTCGACTGAGGAAA
U6	Human	Forward	CTCGCTTCGGCAGCACA
		Reverse	AACGCTTCACGAATTTGCGT
GAPDH	Human	Forward	GAAGGTGAAGGTCGGAGTC
		Reverse	GAAGATGGTGATGGGATTTC

### 2.4 RNase R digestion

A total of 2 μg RNA sample was digested with or without 6 U of RNase R for 30 min at room temperature. The levels of circAGFG1 and AGFG1 were examined by real-time quantitative polymerase chain reaction (RT-qPCR).

### 2.5 Cell transfection

circAGFG1 small-interfering RNA (si-circAGFG1), HIF-1α siRNA (si-HIF-1α), negative control siRNA (si-NC), circAGFG1 overexpression plasmid (circAGFG1), pLCDH-cir (Vector), HIF-1α overexpression plasmid (HIF-1α) and pcDNA, circAGFG1 short hairpin RNA (sh-circAGFG1), sh-NC, miR-28-5p mimic (miR-28-5p), miR-NC, miR-28-5p inhibitor (anti-miR-28-5p), and anti-NC were purchased from Genepharma (Shanghai, China) or Ribobio (Guangzhou, China).

### 2.6 Cell Counting Kit 8 assay

Cell Counting Kit 8 (CCK8) kit (Dojindo, Kumamoto, Japan) was used to detect the proliferation capacity of NSCLC cells. NSCLC cells were seeded into 96-well plates at a density of 5 × 10^3^ cells/well. CCK8 reagent was added to the wells of a 96-well plate after transfection for 0, 24, 48, or 72 h. The plates were placed in the incubator at 37℃ for 2 h followed by the detection of the absorbance value at 450 nm.

### 2.7 Flow cytometry

After appropriate transfection for 72 h, NSCLC cells were resuspended in the binding buffer (BD Biosciences, San Jose, CA, USA) and then simultaneously stained with fluorescein isothiocyanate (FITC)-Annexin V (BD Biosciences) and propidium iodide (PI; BD Biosciences) in the dark for 15 min. The apoptotic NSCLC cells (FITC^+^/PI^+/−^) were identified by the flow cytometry.

### 2.8 Transwell assays

NSCLC cells were starved for 12 h followed by suspending in the culture medium without serum. Cell suspension (100 μL) was added into the upper chambers that were coated (transwell invasion assay) or uncoated (transwell migration assay) with 50 μL Matrigel (Sigma, St. Louis, MO, USA). Subsequently, 600 μL medium with 10% FBS was added into the lower chambers. The transwell plates were placed in the incubator at 37℃ for 24 h, and the metastatic NSCLC cells were dyed with 0.05% gentian violet (Sangon Biotech, Shanghai, China). The number of migrated or invaded NSCLC cells in five random fields was counted and analyzed.

### 2.9 Western blot assay

Proteins were isolated using radioimmunoprecipitation assay (RIPA; Solarbio, Beijing, China) buffer with proteinase inhibitor. Proteins were separated using sodium dodecyl sulfate polyacrylamide gel electrophoresis (SDS-PAGE) gel and then blotted onto the polyvinylidene fluoride (PVDF) membrane (Bio-Rad, Hercules, CA, USA). After blocking with 5% nonfat milk, the membrane was incubated with the following primary antibodies overnight: anti-E-Cadherin (ab1416; Abcam, Cambridge, MA, USA), anti-Vimentin (ab92547; Abcam), anti-N-Cadherin (ab18203; Abcam), anti-HIF-1α (ab16066; Abcam), anti-glucose transporter 1 (anti-GLUT1; ab40084; Abcam), anti-phosphoglycerate kinase 1 (anti-PGK1; ab199438; Abcam), anti-pyruvate kinase M2 (anti-PKM2; ab137852; Abcam), and GAPDH (ab181602, Abcam) were used as the internal references for immunoblot. Appropriate horseradish peroxidase (HRP)-conjugated secondary antibodies (Abcam) were used to incubate with the membrane the next day. Protein signals were visualized with the enhanced chemiluminescence detection kit (Millipore, Billarica, MA, USA).

### 2.10 Establishment of circAGFG1/miR-28-5p/HIF-1α axis

StarBase online database was used to predict the targets of circAGFG1 and miR-28-5p on the basis of the complementary sites between the putative targets and circAGFG1 or miR-28-5p.

### 2.11 Dual-luciferase reporter assay

The partial sequence of circAGFG1 or HIF-1α, containing miR-28-5p-binding sites, was inserted into the pGL3 plasmid (Ambion, Austin, TX, USA), termed as circAGFG1 WT or HIF-1α WT. An altered sequence in circAGFG1 or HIF-1α, harboring the mutant type binding sites with miR-28-5p, was also cloned into the pGL3 plasmid (Ambion), termed as circAGFG1 MUT or HIF-1α MUT. After transfecting these luciferase plasmids (120 ng) along with miR-28-5p or miR-NC (40 nM) for 48 h, the luciferase intensities were examined using the Dual-Luciferase Reporter Assay System (Promega, Madison, WI, USA).

### 2.12 RNA immune co-precipitation (RIP) assay

NSCLC cells were lysed using RIP lysis buffer (Bio-Rad). Protein-A Sepharose beads (Bio-Rad) were precoated with Argonaute 2 antibody (anti-Ago2; Bio-Rad) or immunoglobulin G antibody (anti-IgG; Bio-Rad). The cell lysate was incubated with the precoated Protein-A Sepharose beads for 3 h at 4℃. RNA was extracted using TRIzol reagent (Takara) and detected by RT-qPCR.

### 2.13 Glycolytic analysis

The production of lactate and ATP and the uptake of glucose were assessed through using Lactate Assay Kit II (Biovision, Milpitas, CA, USA), ATP Colorimetric Assay kit (Biovision), and Glucose Uptake Colorimetric Assay kit (Biovision).

### 2.14 Xenograft tumor model

A total of 14 immunodeficient nude mice were housed in 40–70% humidity at 20–26℃. These nude mice were arbitrarily divided into the sh-NC group (*n* = 7) and sh-circAGFG1 group (*n* = 7). A549 cells stably expressing sh-NC or sh-circAGFG1 were injected into the right side of the back of mice (2 × 10^6^/100 μL phosphate-buffered saline [PBS]). The tumor volume was continuously monitored every week, and the tumor volume was analyzed by (length × width^2^)/2. This study was authorized by the Institutional Animal Care and Use Committee of No. 215 Hospital of Shaanxi Nuclear Industry.

### 2.15 Statistical analysis

Data were represented as mean ± standard deviation (SD). Differences in two groups were analyzed by Student’s *t*-test, and one-way analysis of variance (ANOVA) followed by Tukey’s test was used to assess the comparison in multiple groups. The survival curve of NSCLC patients was generated by the Kaplan-Meier plot and the log-rank test. The receiver-operating characteristic (ROC) curve was analyzed to measure the area under the curve (AUC) value for circAGFG1 expression in tumor tissues and adjacent normal tissues. *P* < 0.05 was identified as statistically significance.

## 3 Results

### 3.1 circAGFG1 is upregulated in NSCLC

**Figure 1:**
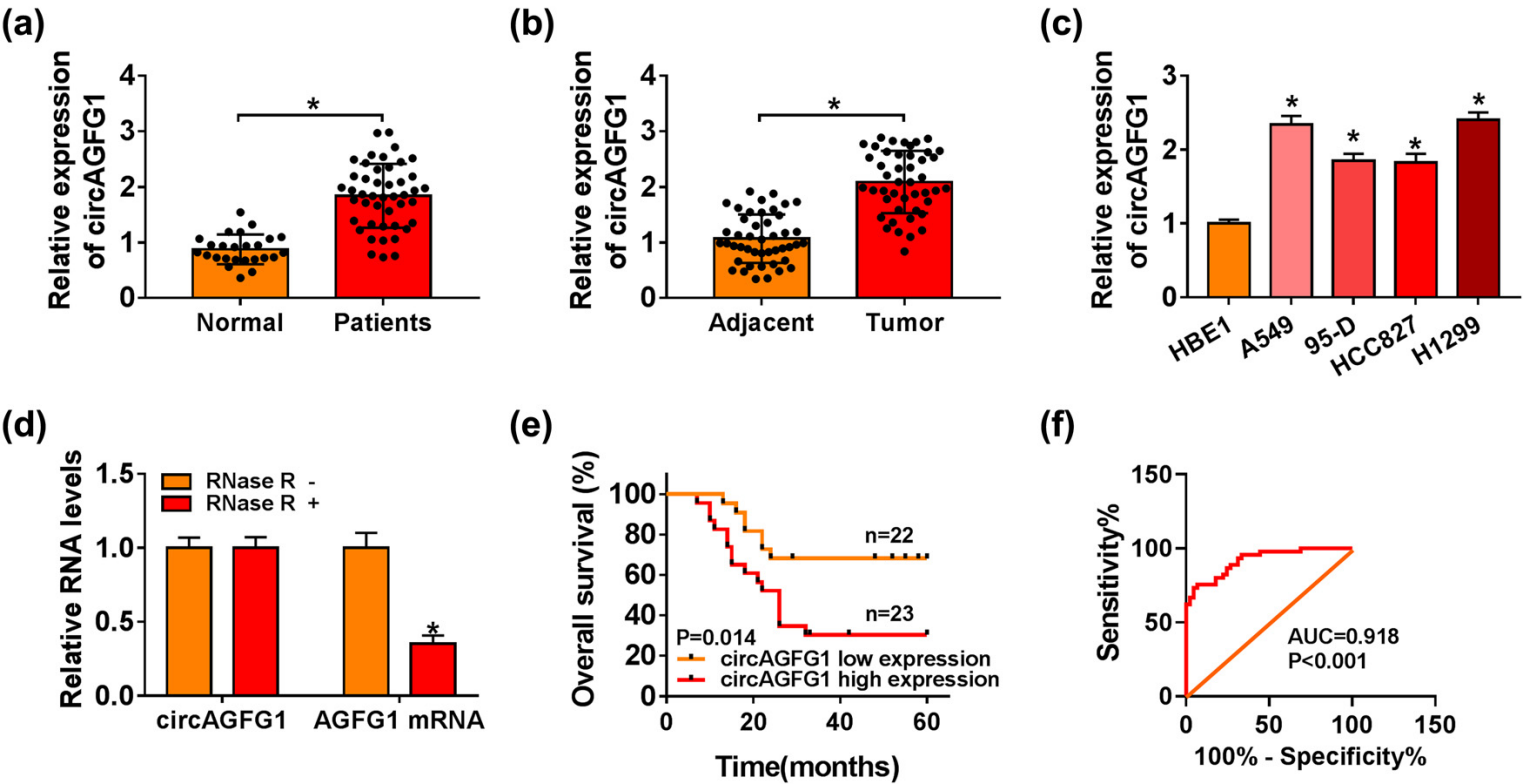
circAGFG1 is upregulated in NSCLC. (a) circAGFG1 expression in the serum samples of NSCLC patients (*n* = 45) and healthy controls (*n* = 25) was examined by RT-qPCR. (b) RT-qPCR was implemented to detect the expression of circAGFG1 in NSCLC tumor tissues (*n* = 45) and adjacent normal tissues (*n* = 45). (c) circAGFG1 abundance in a human bronchial epithelial cell line (HBE1) and four NSCLC cell lines (A549, 95-D, HCC827, and H1299) were examined by RT-qPCR. (d) RNA samples from A549 cells were equally divided into two parts, and then, these two parts of RNA samples were digested with RNase R or not, and the expression of circAGFG1 and AGFG1 mRNA was analyzed by RT-qPCR. (e) NSCLC patients were divided into circAGFG1 high expression group (*n* = 23) and low expression group (*n* = 22) based on the median value of circAGFG1 expression. The overall survival curves of the two groups were generated. (f) ROC curve was analyzed to assess the diagnostic value of circAGFG1 expression in NSCLC patients. *P < 0.05.

We first measured the expression profile of circAGFG1 in NSCLC. We collected serum samples of NSCLC patients (*n* = 45) and healthy volunteers (*n* = 25) to examine the expression of circAGFG1. As displayed in Figure 1a, there was an obvious upregulation in circAGFG1 expression in the serum samples from NSCLC patients. Moreover, the tumor tissues (*n* = 45) and adjacent normal tissues (*n* = 45) from a total of 45 NSCLC patients were collected to analyze circAGFG1 expression. circAGFG1 was notably upregulated in NSCLC tumor tissues in comparison with that in adjacent normal tissues (Figure 1b). A panel of NSCLC cell lines, including A549, 95-D, HCC827, and H1299, along with the human bronchial epithelial cell line (HBE1) were used to analyze circAGFG1 expression. circAGFG1 abundance was significantly enhanced in all NSCLC cell lines compared with HBE1 cell line (Figure 1c), and A549 and H1299 cell lines were chosen for further analysis on account of their higher expression of circAGFG1. We confirmed the circular structure and the stability of circAGFG1 using RNase R. As displayed in Figure 1d, RNase R treatment degraded AGFG1 mRNA, and the level of circAGFG1 remained almost unchanged with or without RNase R treatment. NSCLC patients with high expression of circAGFG1 were associated with the low survival rate compared with who had low expression of circAGFG1 (Figure 1e). The ROC curve was used to analyze the diagnostic value of circAGFG1 expression in NSCLC patients. The data showed that AUC reached 0.918 (Figure 1f). Taken together, circAGFG1 was aberrantly upregulated in NSCLC, and it might be a poor prognosis indicator for NSCLC patients.

### 3.2 circAGFG1 silencing restrains the proliferation and metastasis and promotes the apoptosis of NSCLC cells

**Figure 2:**
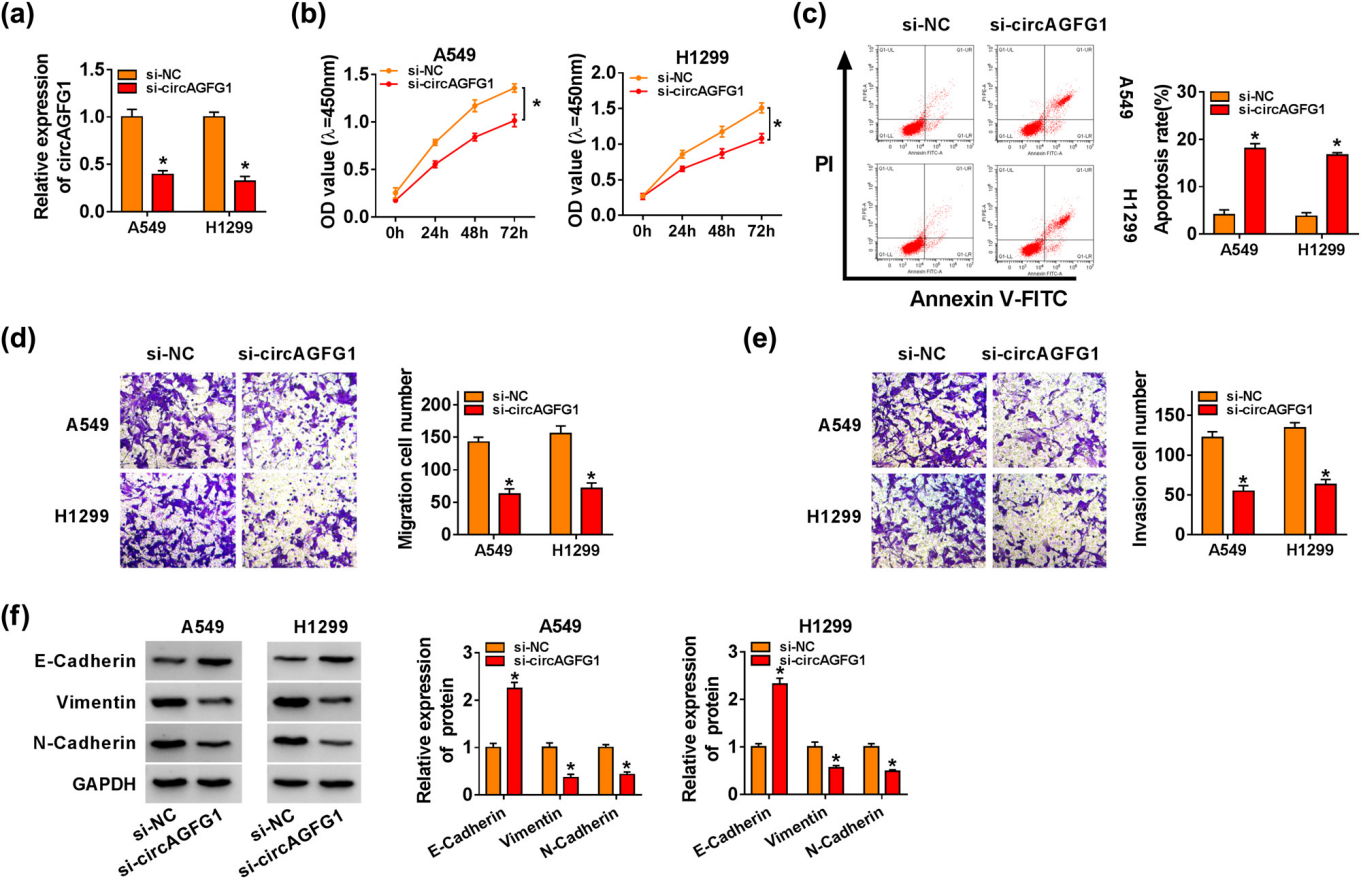
circAGFG1 silencing restrains the proliferation and metastasis and promotes the apoptosis of NSCLC cells. (a–f) NSCLC cells were transfected with si-NC or si-circAGFG1. (a) circAGFG1 abundance was examined in A549 and H1299 cells by RT-qPCR. (b) CCK8 assay was utilized to assess the proliferation ability of NSCLC cells. (c) Flow cytometry was used to evaluate the apoptosis of NSCLC cells. (d and e) Transwell assays were conducted to assess the migration and invasion abilities of NSCLC cells. Magnification: 100×. (f) The expression of metastasis-associated protein markers (E-Cadherin, Vimentin, and N-Cadherin) was analyzed by the western blot assay. *P < 0.05.

We conducted loss-of-function experiments in NSCLC cells using si-circAGFG1 or its control si-NC. Si-circAGFG1 transfection markedly reduced the expression of circAGFG1 in NSCLC cells (Figure 2a). Cell proliferation was restrained with the interfering of circAGFG1 in NSCLC cells (Figure 2b). The apoptotic NSCLC cells (FITC^+^/PI^+/−^) were analyzed in the si-NC transfected group and the si-circAGFG1 transfected group. As displayed in Figure 2c, circAGFG1 interference elevated the apoptosis rate of NSCLC cells. The migration and invasion abilities of transfected NSCLC cells were assessed by transwell migration and invasion assays. With the silencing of circAGFG1, the number of both migrated and invaded NSCLC cells was decreased (Figure 2d and e), suggesting that circAGFG1 silencing restrained the migration and invasion of NSCLC cells. The levels of metastasis-related markers (E-Cadherin, Vimentin, and N-Cadherin) were measured by the western blot assay to verify the influence of circAGFG1 silencing on the metastasis of NSCLC cells. The expression of Vimentin and N-Cadherin was reduced, while the level of E-Cadherin was increased in the circAGFG1 interference group when compared with the si-NC group (Figure 2f). Overall, circAGFG1 knockdown hampered the proliferation and metastasis and induced the apoptosis of NSCLC cells.

### 3.3 miR-28-5p interacts with circAGFG1 in NSCLC cells

**Figure 3:**
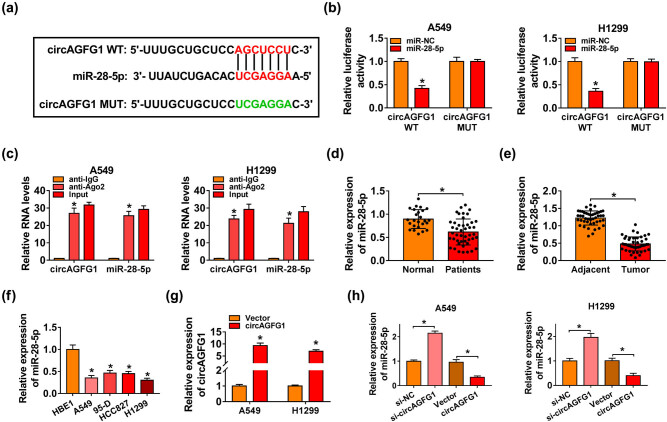
miR-28-5p interacts with circAGFG1 in NSCLC cells. (a) The predicted binding sites between circAGFG1 and miR-28-5p via StarBase software were shown in red, and the mutant binding sequence with miR-28-5p in circAGFG1 was shown in green. (b) A dual-luciferase reporter assay was performed to confirm the binding relationship between miR-28-5p and circAGFG1 in NSCLC cells. (c) RIP assay was implemented to confirm the target relationship between miR-28-5p and circAGFG1 in NSCLC cells. (d) RT-qPCR assay was conducted to analyze the expression of miR-28-5p in the serum samples of NSCLC patients (*n* = 45) and healthy volunteers (*n* = 25). (e) miR-28-5p expression in NSCLC tissues and adjacent nontumor tissues was analyzed by RT-qPCR. (f) RT-qPCR was applied to analyze the expression of miR-28-5p in HBE1 cell line and four NSCLC cell lines. (g) The expression of circAGFG1 was detected in A549 and H1299 cells transfected with Vector or circAGFG1 by RT-qPCR. (h) A549 and H1299 cells were transfected with si-NC, si-circAGFG1, vector, or circAGFG1. The level of miR-28-5p in transfected NSCLC cells was measured by RT-qPCR. *P < 0.05.

StarBase database was used to explore circAGFG1-miRNAs binding relationships. The putative complementary sites between circAGFG1 and miR-28-5p were shown in red (Figure 3a). The binding sites in circAGFG1 were mutated by “UCGAGGA” (Figure 3a, shown in green) to perform a dual-luciferase reporter assay to test if “AGCUCCU” in circAGFG1 was involved the interaction between miR-28-5p and circAGFG1. miR-28-5p overexpression significantly reduced the luciferase activity in circAGFG1 WT group than that in miR-NC and circAGFG1 WT groups (Figure 3b), suggesting the binding relationship between miR-28-5p and circAGFG1 in NSCLC cells. To test if “AGCUCCU” in circAGFG1 was involved in the interaction between miR-28-5p and circAGFG1, we constructed mutant type luciferase reporter plasmid, named as circAGFG1 MUT. The luciferase activity remained unchanged in the circAGFG1 MUT group when co-transfected with miR-NC or miR-28-5p (Figure 3b), suggesting that “AGCUCCU” sites in circAGFG1 were the binding sites between circAGFG1 and miR-28-5p. Ago2 was a core component in the RNA-induced silence complex (RISC), which contained miRNAs, and we subsequently tested if circAGFG1 bound to miR-28-5p in RISC using Ago2 antibody. As displayed in Figure 3c, both circAGFG1 and miR-28-5p were enriched in the anti-Ago2 group compared with the anti-IgG group. miR-28-5p was downregulated in the serum samples of NSCLC patients in contrast to that in the serum samples of healthy volunteers (Figure 3d). Also, miR-28-5p was notably downregulated in NSCLC tissues compared with adjacent normal tissues (Figure 3e). Compared with the HBE1 cell line, miR-28-5p expression was significantly reduced in four NSCLC cell lines (Figure 3f). The overexpression efficiency of circAGFG1 was high in NSCLC cells (Figure 3g). circAGFG1 interference upregulated miR-28-5p expression in NSCLC cells, while circAGFG1 overexpression caused a reduction in miR-28-5p level (Figure 3h). Taken together, miR-28-5p was a target of circAGFG1 in NSCLC cells.

### 3.4 circAGFG1 enhances the malignant behaviors of NSCLC cells through targeting miR-28-5p

**Figure 4:**
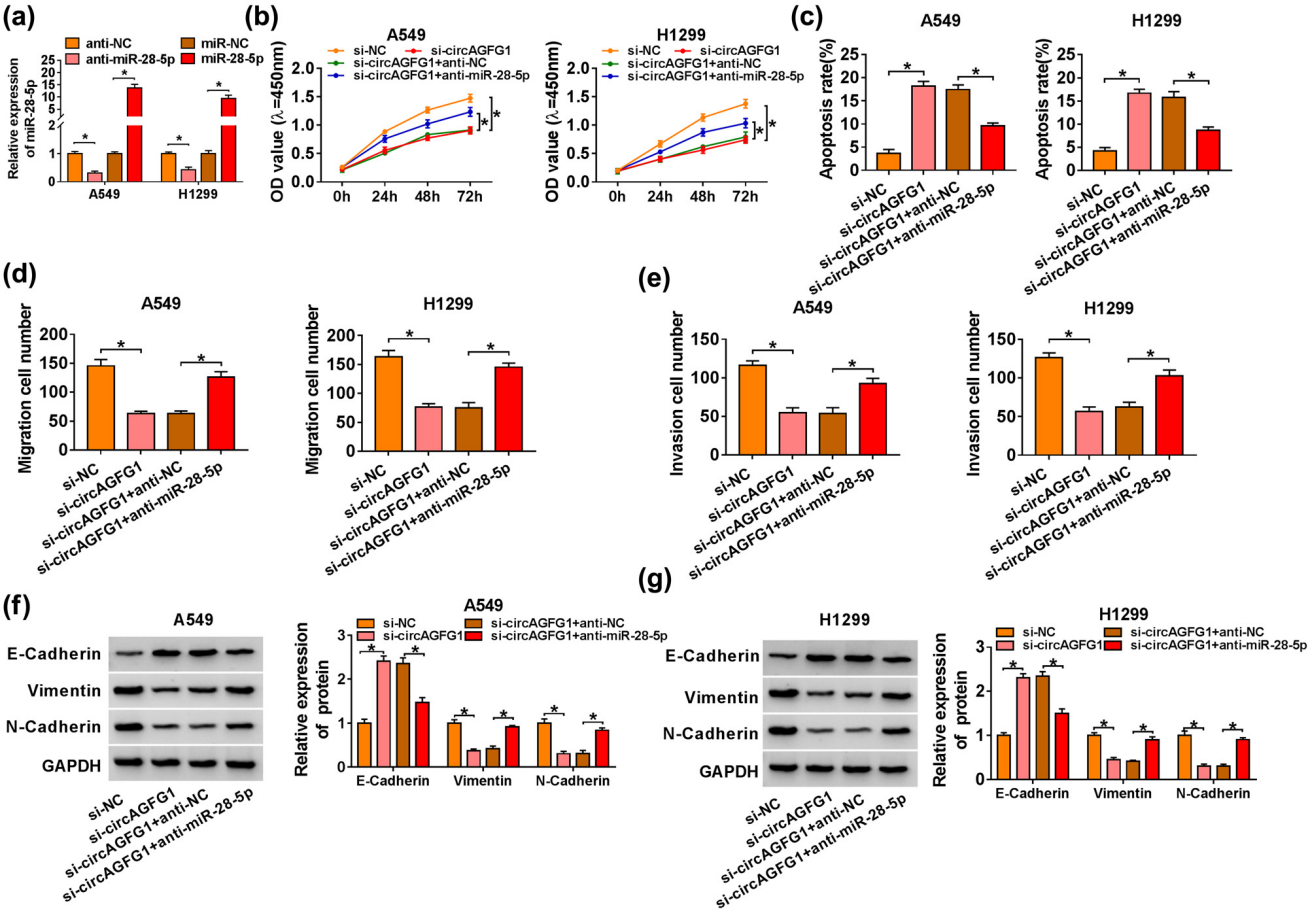
circAGFG1 enhances the malignant behaviors of NSCLC cells through targeting miR-28-5p. (a) The transfection efficiency of anti-miR-28-5p in NSCLC cells was assessed by RT-qPCR. (b–g) A549 and H1299 cells were transfected with si-NC, si-circAGFG1, si-circAGFG1 + anti-NC, or si-circAGFG1 + anti-miR-28-5p. (b) CCK8 assay was utilized to assess the proliferation of NSCLC cells. (c) The apoptosis rate in different groups was analyzed by flow cytometry. (d and e) The abilities of migration and invasion in NSCLC cells were analyzed by transwell migration and invasion assays. (f and g) Western blot assay was carried out to examine the protein levels of E-Cadherin, Vimentin, and N-Cadherin in NSCLC cells. *P < 0.05.

**Figure S1:**
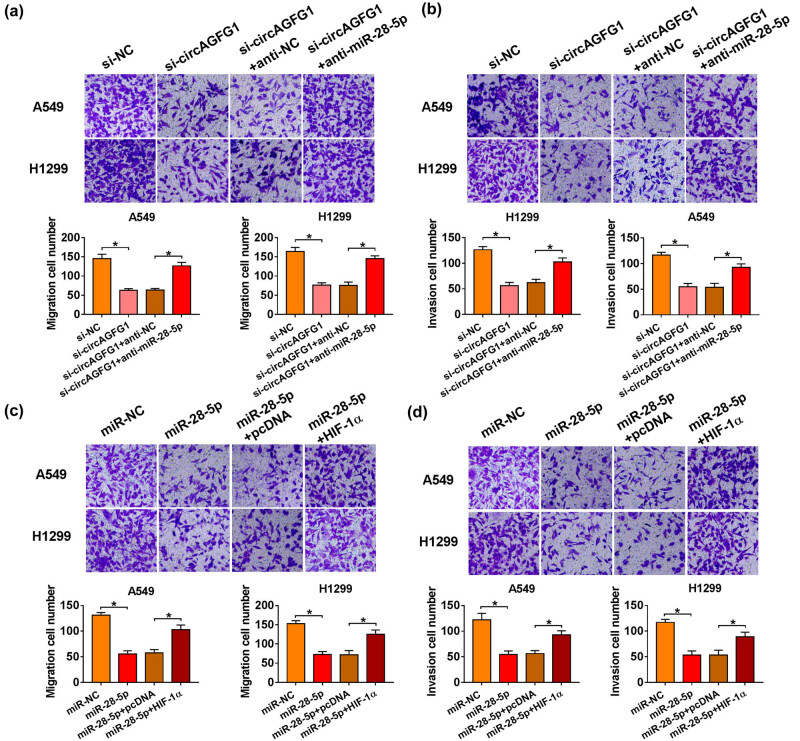
The supplementary images of transwell migration and invasion assays. (a and b) The supplementary images of transwell assays in Figure 4d and e. (c and d) The supplementary images of transwell assays in Figure 6c and d. *P < 0.05.

The transfection of anti-miR-28-5p notably reduced miR-28-5p expression in A549 and H1299 cells (Figure 4a). We conducted rescue experiments to disclose whether circAGFG1 functioned through targeting miR-28-5p. A549 and H1299 cells were transfected with si-NC, si-circAGFG1, si-circAGFG1 + anti-NC, or si-circAGFG1 + anti-miR-28-5p. circAGFG1 silencing-mediated suppressive effect on the proliferation of NSCLC cells was partially restored by the addition of anti-miR-28-5p (Figure 4b). circAGFG1 interference triggered the apoptosis of NSCLC cells, and this promoting influence was partly counteracted in si-circAGFG1 and anti-miR-28-5p co-transfected groups (Figure 4c). The migration and invasion abilities of NSCLC cells were largely rescued with the addition of anti-miR-28-5p (Figure 4d and e, Figure S1a and b). circAGFG1 silencing-mediated influences on the levels of E-Cadherin, Vimentin, and N-Cadherin were largely overturned by the introduction of anti-miR-28-5p in NSCLC cells (Figure 4f and g). Overall, circAGFG1 promoted NSCLC progression partly through sponging miR-28-5p.

### 3.5 miR-28-5p interacts with the 3′ untranslated region of HIF-1α in NSCLC cells

**Figure 5:**
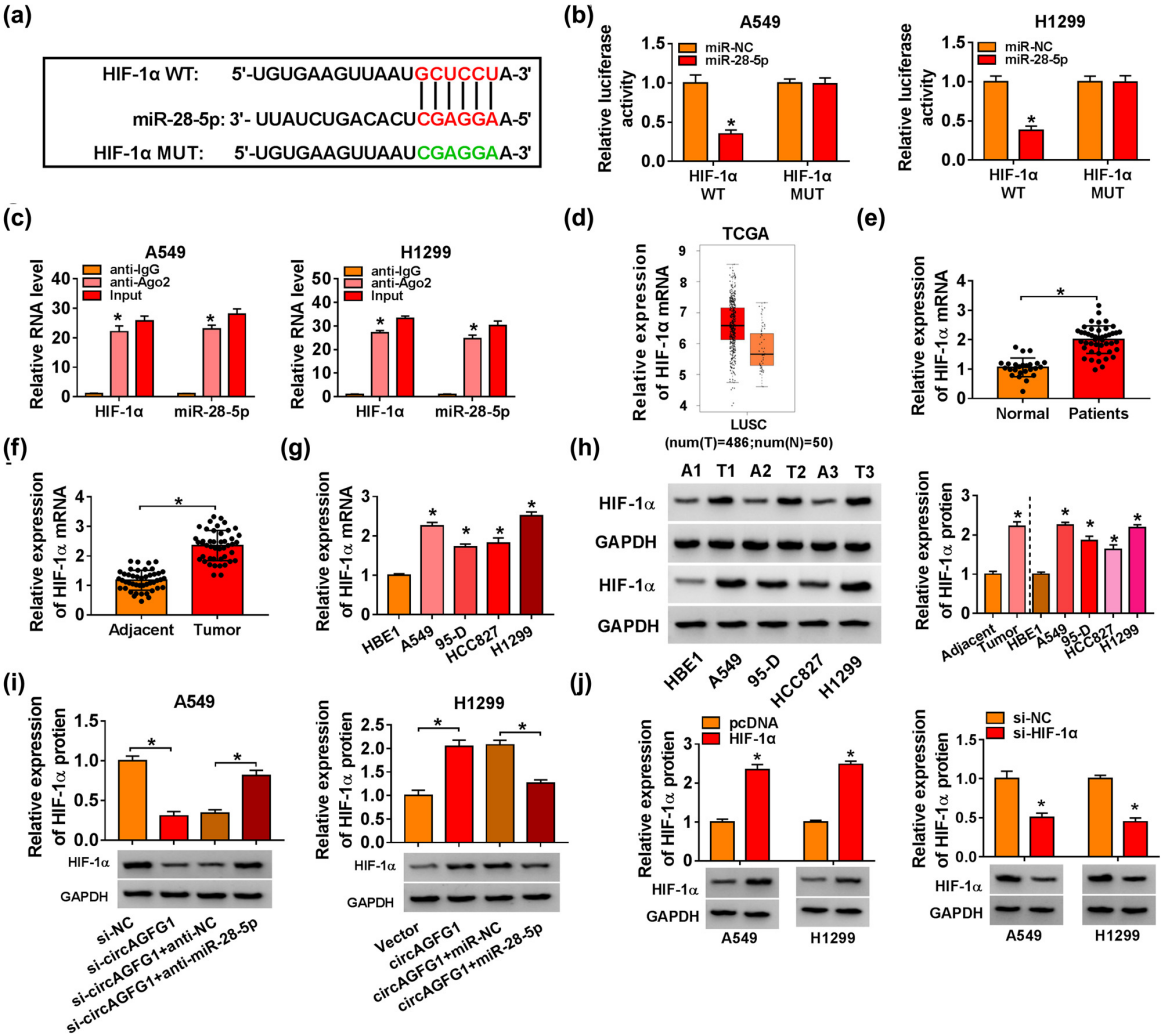
miR-28-5p interacts with the 3′ untranslated region (3′UTR) of HIF-1α in NSCLC cells. (a) HIF-1α was verified as a possible target of miR-28-5p by StarBase online database. (b) A Dual-luciferase reporter assay was conducted to analyze the target interaction between miR-28-5p and HIF-1α in NSCLC cells. (c) The target interaction between miR-28-5p and HIF-1α in NSCLC cells was tested by RIP assay. (d) The expression of HIF-1α in lung squamous cell carcinoma (LUSC) tissues (*n* = 486) and normal tissues (*n* = 50) according to the data of the TCGA database (http://gepia.cancer-pku.cn/) was analyzed. (e) HIF-1α mRNA expression in NSCLC serum samples (*n* = 45) and healthy serum samples (*n* = 25) was analyzed by RT-qPCR. (f) RT-qPCR was implemented to detect the mRNA expression of HIF-1α in NSCLC tumor specimens (*n* = 45) and adjacent normal specimens (*n* = 45). (g) The mRNA level of HIF-1α was measured in HBE1, A549, 95-D, HCC827, and H1299 cells by RT-qPCR. (h) Western blot assay was applied to analyze the protein level of HIF-1α in NSCLC tissues, adjacent normal tissues, HBE1 cell line, and four NSCLC cell lines. (i) A549 cells were transfected with miR-NC, miR-28-5p, miR-28-5p + vector, or miR-28-5p + circAGFG1. H1299 cells were transfected with anti-NC, anti-miR-28-5p, anti-miR-28-5p + si-NC, or anti-miR-28-5p + si-circAGFG1. The protein expression of HIF-1α was examined in NSCLC cells by the western blot assay. (j) The overexpression efficiency of HIF-1α overexpression plasmid was analyzed in NSCLC cells by RT-qPCR. *P < 0.05.

**Figure S2:**
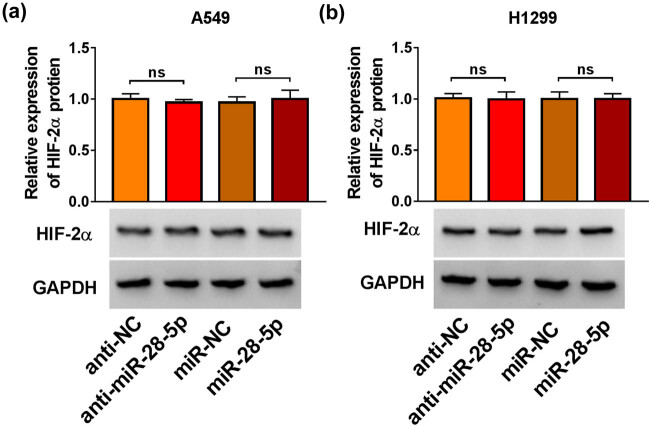
MiR-28-5p overexpression or silencing has no effect on HIF-2α expression in NSCLC cells. (a and b) RT-qPCR was applied to determine the expression of HIF-2α in NSCLC cells with the overexpression or silencing of miR-28-5p.

HIF-1α was predicted as a possible target of miR-28-5p by StarBase database. The complementary sites between miR-28-5p and HIF-1α are shown in Figure 5a. A549 and H1299 cells were co-transfected with miR-NC or miR-28-5p and HIF-1α WT or HIF-1α MUT to perform the dual-luciferase reporter assay. miR-28-5p transfection significantly decreased the luciferase activity in the HIF-1α WT group compared with that in the HIF-1α MUT group (Figure 5b), demonstrating that miR-28-5p bound to HIF-1α in NSCLC cells. The results of the RIP assay demonstrated that both miR-28-5p and HIF-1α were enriched in the anti-Ago2 group compared with that in the anti-IgG group (Figure 5c), suggesting the target interaction between miR-28-5p and HIF-1α in NSCLC cells. According to the data from TCGA database, HIF-1α mRNA was elevated in lung squamous cell carcinoma (LUSC) tissues (*n* = 486) compared with normal tissues (*n* = 50) (Figure 5d). Moreover, HIF-1α mRNA was also enhanced in the serum samples and tumor tissues of NSCLC patients compared with their matching controls (Figure 5e and f). HIF-1α mRNA was also elevated in NSCLC cell lines compared with that in HBE1 cell line (Figure 5g). The protein expression of HIF-1α was also upregulated in NSCLC tissues and cell lines in comparison with that in adjacent normal tissues and HBE1 cell line via the western blot assay (Figure 5h). We transfected A549 cells with miR-28-5p alone or together with circAGFG1 to explore the regulatory relationship between circAGFG1 and HIF-1α. miR-28-5p overexpression reduced the protein expression of HIF-1α, and the addition of circAGFG1 overexpression plasmid recovered the protein level of HIF-1α (Figure 5i). H1299 cells were transfected with anti-miR-28-5p or together with si-circAGFG1 to further confirm the regulatory relationship between circAGFG1 and HIF-1α in NSCLC cells. miR-28-5p interference elevated the expression of HIF-1α, and the introduction of si-circAGFG1 reduced the expression of HIF-1α (Figure 5i). In addition to HIF-1α, we also assessed the target interaction and the regulatory relationship between miR-28-5p and HIF-2α. On the basis of the results of bioinformatic databases, including TargetScan and StarBase, there was no putative binding sequence between miR-28-5p and HIF-2α. Furthermore, the results of the western blot assay revealed that the overexpression or silencing of miR-28-5p had no influence on HIF-2α expression in NSCLC cells (Figure S2a and b). Hence, we selected HIF-1α for further experiment. The overexpression efficiency of HIF-1α plasmid was high in NSCLC cells (Figure 5j). These findings together demonstrated that circAGFG1 enhanced the enrichment of HIF-1α via sponging miR-28-5p in NSCLC cells.

### 3.6 miR-28-5p suppresses the malignant potential of NSCLC cells via targeting HIF-1α

**Figure 6:**
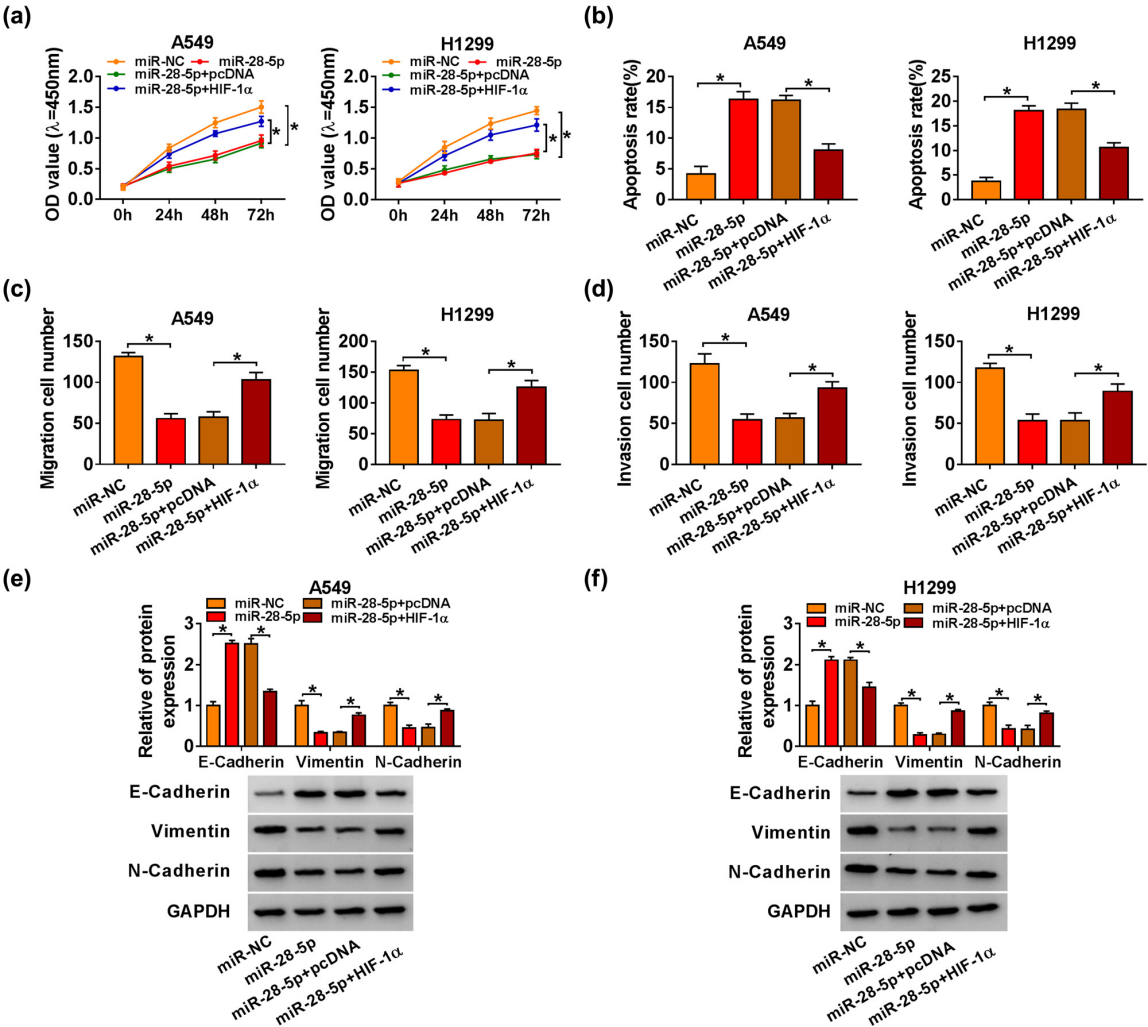
miR-28-5p suppresses the malignant potential of NSCLC cells via targeting HIF-1α. (a–f) A549 and H1299 cells were transfected with miR-NC, miR-28-5p, miR-28-5p + pcDNA, or miR-28-5p + HIF-1α. (a) CCK8 assay was used to evaluate the proliferation ability of NSCLC cells. (b) The apoptosis of NSCLC cells was analyzed by flow cytometry. (c) A Transwell migration assay was conducted to measure the migration of transfected NSCLC cells. (d) The invasion ability of NSCLC cells was detected by the transwell invasion assay. (e and f) The protein abundance of E-Cadherin, Vimentin, and N-Cadherin in NSCLC cells was analyzed by the western blot assay. *P < 0.05.

miR-28-5p overexpression suppressed the proliferation, migration, and invasion and induced the apoptosis of NSCLC cells (Figure 6a–f). The proliferation ability of NSCLC cells was partly recovered in miR-28-5p and HIF-1α co-transfected group (Figure 6a). miR-28-5p-induced apoptosis in NSCLC cells was attenuated by the addition of HIF-1α (Figure 6b). The results of transwell assays revealed that HIF-1α addition alleviated miR-28-5p overexpression-mediated inhibitory effects on the migration and invasion of NSCLC cells (Figure 6c and d, Figure S1c and d). HIF-1α introduction also reversed the influence of miR-28-5p overexpression on the expression of E-Cadherin, Vimentin, and N-Cadherin in NSCLC cells (Figure 6e and f). Taken together, miR-28-5p suppressed the proliferation, migration, and invasion and accelerated the apoptosis of NSCLC cells through targeting HIF-1α.

### 3.7 circAGFG1 silencing suppresses cell glycolytic metabolism partly through targeting miR-28-5p/HIF-1α axis

**Figure 7:**
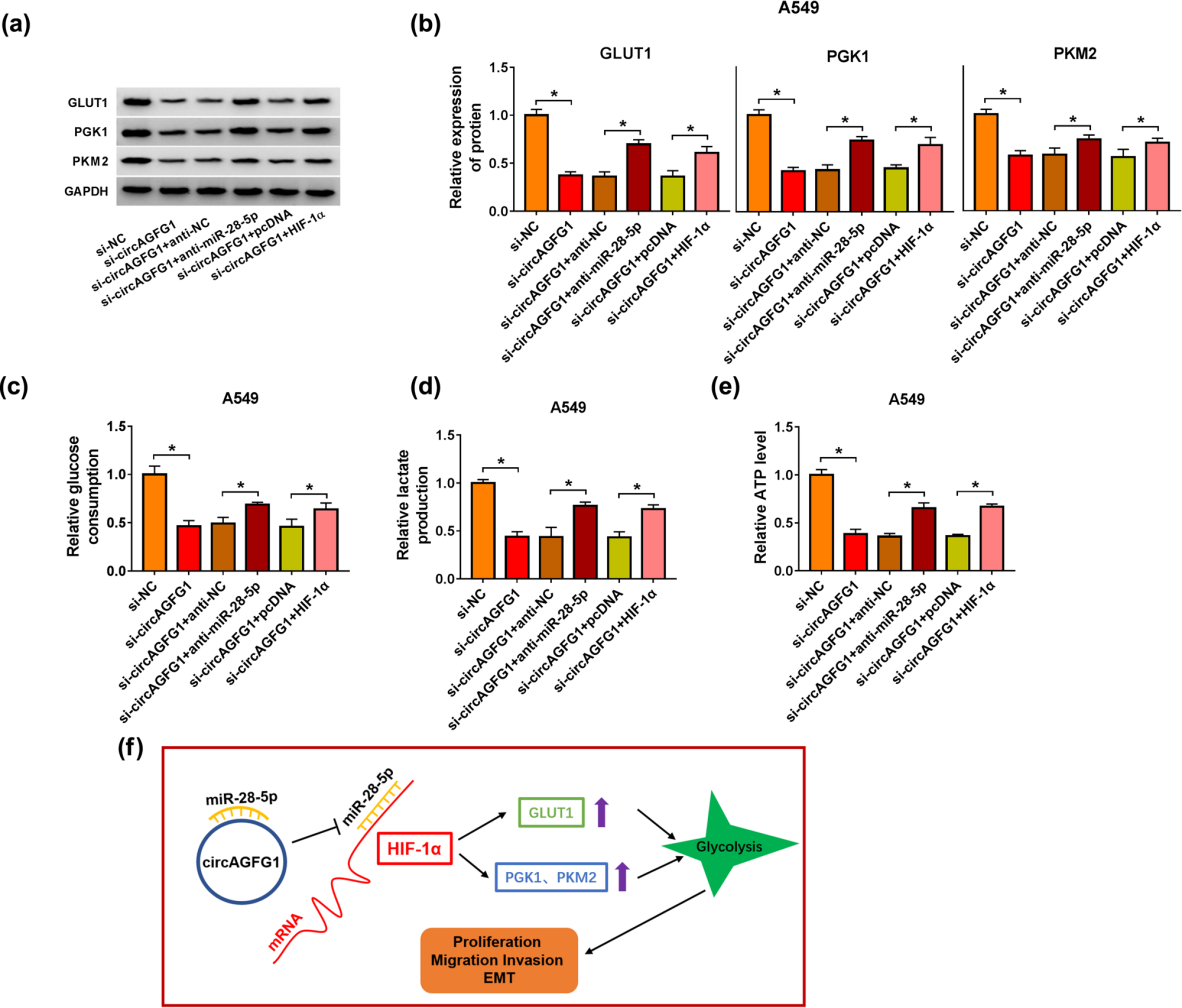
circAGFG1 silencing suppresses cell glycolytic metabolism partly through targeting the miR-28-5p/HIF-1α axis. (a–e) A549 cells were divided into six groups: si-NC, si-circAGFG1, si-circAGFG1 + anti-NC, si-circAGFG1 + anti-miR-28-5p, si-circAGFG1 + pcDNA, and si-circAGFG1 + HIF-1α. (a and b) The expression of glycolysis-related proteins (GLUT1, PGK1, and PKM2) was examined by the western blot assay. (c–e) The uptake of glucose, the production of lactate, and the level of ATP were analyzed in A549 cells using their corresponding kits. (f) A schematic diagram of the working mechanism behind the oncogenic role of circAGFG1 in NSCLC cells was displayed. *P < 0.05.

To test if circAGFG1/miR-28-5p/HIF-1α axis was involved in the regulation of cellular glycolytic metabolism, we divided A549 into six groups: si-NC, si-circAGFG1, si-circAGFG1 + anti-NC, si-circAGFG1 + anti-miR-28-5p, si-circAGFG1 + pcDNA, and si-circAGFG1 + HIF-1α. The protein expression of glycolysis-related markers (GLUT1, PGK1, and PKM2) in A549 cells was measured by the western blot assay. circAGFG1 silencing reduced the protein expression of GLUT1, PGK1, and PKM2, while the levels of these glycolysis-associated proteins were partly recovered with the interference of miR-28-5p or the overexpression of HIF-1α (Figure 7a and b). Subsequently, we analyzed the consumption of glucose, lactate production, and ATP level in transfected A549 cells. circAGFG1 knockdown-mediated suppressive effects on the consumption of glucose and the production of lactate and ATP were all partly reversed by the silencing of miR-28-5p or the overexpression of HIF-1α (Figure 7c–e). To sum up, circAGFG1 promoted the malignant behaviors of NSCLC cells through promoting the glycolysis partly via targeting the miR-28-5p/HIF-1α axis (Figure 7f).

### 3.8 circAGFG1 knockdown blocks the NSCLC tumor growth *in vivo*

**Figure 8:**
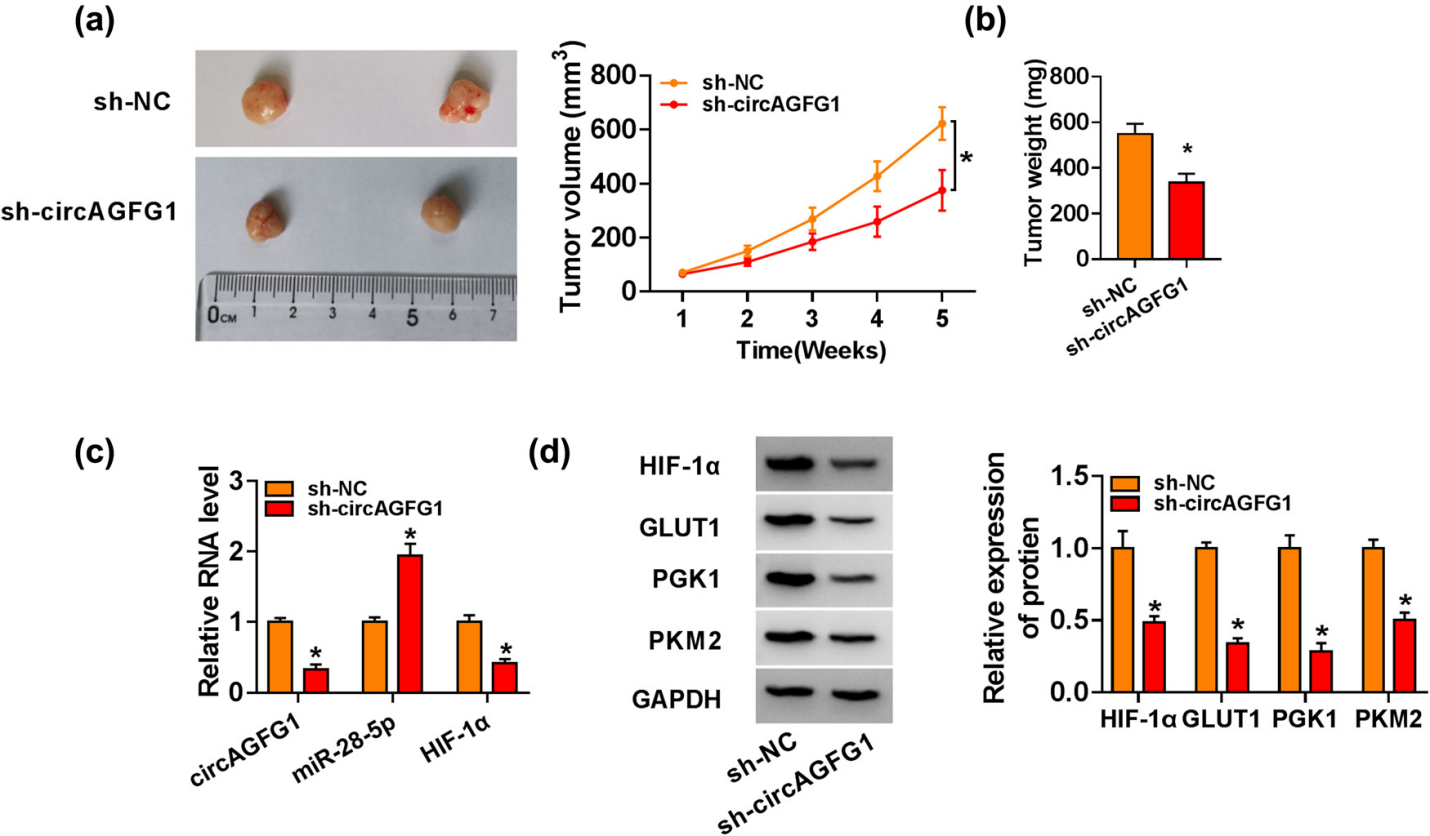
circAGFG1 knockdown blocks the NSCLC tumor growth *in vivo*. (a) Tumor dimension in sh-NC group and sh-circAGFG1 group was recorded every week. (b) Tumors in the sh-NC group and the sh-circAGFG1 group were weighed after 5-week inoculation. (c) RT-qPCR was applied to detect the expression of circAGFG1, miR-28-5p, and HIF-1α in tumor tissues. (d) Western blot assay was conducted to detect the protein expression of HIF-1α, GLUT1, PGK1, and PKM2 in tumor tissues. *P < 0.05.

Xenograft tumor model was established through using A549 cell line stably expressing sh-NC or sh-circAGFG1. The tumor growth curve was generated through continuously monitoring the tumor volume for 5 weeks. After inoculation for 5 weeks, tumors were dissected and weighed. As displayed in Figure 8a and b, circAGFG1 knockdown reduced the growth of NSCLC tumors. The expression of molecules in the circAGFG1/miR-28-5p/HIF-1α axis and glycolytic metabolism was detected by RT-qPCR or the western blot assay. The abundance of circAGFG1 and HIF-1α mRNA was markedly decreased in tumor tissues of the sh-circAGFG1 group compared with that of the sh-NC group (Figure 8c). The expression of miR-28-5p exhibited an opposite trend to circAGFG1 or HIF-1α (Figure 8c). circAGFG1 silencing also reduced the protein expression of HIF-1α and glycolysis-related markers (GLUT1, PGK1, and PKM2) in tumor tissues (Figure 8d). Taken together, circAGFG1 interference reduced the NSCLC tumor growth *in vivo*.

## 4 Discussion

circRNAs are identified as powerful regulators in many cancers [21]. circRNAs act as oncogenes or tumor suppressors to sponge miRNAs to regulate cellular behaviors, thereby regulating the progression of diverse cancers, including NSCLC [22]. For instance, circ_0001649 blocked the development of NSCLC through sponging miR-331-3p and miR-338-5p [23]. circ_0002483 suppressed the development of NSCLC and elevated the chemosensitivity of NSCLC cells to Taxol through sponging miR-182-5p [24]. We found that circAGFG1 was upregulated in NSCLC. According to the data of the survival curve analysis, we found that high expression of circAGFG1 might be a marker of poor prognosis of NSCLC patients. The oncogenic role of circAGFG1 in triple-negative breast cancer, cervical cancer, and NSCLC has been reported in previous studies. Yang et al. found that circAGFG1 accelerated the progression of triple-negative breast cancer through targeting the miR-195-5p/CCNE1 axis [6]. Wu and Zhou demonstrated that circAGFG1 facilitated the development of cervical cancer through targeting the miR-370-3p/RAF1 axis [5]. Xue et al. reported that circAGFG1 accelerated the metastasis of NSCLC cells through upregulating ZNF281 via sponging miR-203 [7]. Consistent with the previous studies, the results of loss-of-function experiments demonstrated that circAGFG1 promoted the proliferation, migration, and invasion while restrained the apoptosis of NSCLC cells.

The dysregulated miRNAs are implicated in the pathogenesis of many malignancies [25,26]. For example, miR-4458 expression was reduced in breast cancer tissues and cell lines, and miR-4458 suppressed the proliferation and motility of breast cancer cells via CPSF4 [27]. The miRNA targets of circAGFG1 were sought using StarBase database. The interaction between circAGFG1 and miR-28-5p in NSCLC cells was then confirmed. Long noncoding RNA (lncRNA) UCA1 promoted colon cancer progression through sequestering miR-28-5p to elevate the HOXB3 level [28]. Liu et al. demonstrated that LOXL1-AS1 promoted the proliferation and migration of pancreatic cancer cells through upregulating SEMA7A via sponging and suppressing miR-28-5p [12]. Consistent with the previous studies, we found that the suppressive effects of circAGFG1 interference on the malignant behaviors of NSCLC cells were largely alleviated by the addition of anti-miR-28-5p, suggesting that the suppressive influences of circAGFG1 silencing on the malignant behaviors of NSCLC cells were partly based on its negative regulatory relationship with miR-28-5p.

HIF-1 is composed of constitutively expressed HIF-1β subunit and hypoxic-responsive HIF-1α subunit [29]. HIF-1 signal pathway is implicated in the regulation of cellular hypoxic response, cell cycle progression, and glycolytic metabolism [30,31]. HIF-1α promoted the glycolytic metabolism to elevate cell viability under hypoxia [31]. The target relationship between HIF-1α and miR-28-5p was verified, and miR-28-5p suppressed malignant behaviors of NSCLC cells partly through targeting and suppressing HIF-1α. We also assessed the effect of circAGFG1/miR-28-5p/HIF-1α axis on the glycolysis of NSCLC cells. circAGFG1 silencing downregulated the expression of glycolytic crucial enzymes (GLUT1, PGK1, and PKM2) and reduced the uptake of glucose and the production of lactate and ATP, and the silencing of miR-28-5p or the overexpression of HIF-1α largely rescued the glycolysis of NSCLC cells, suggesting that circAGFG1 accelerated the glycolysis of NSCLC cells partly through miR-28-5p/HIF-1α axis. Given the data that circAGFG1 enhanced the malignant potential of NSCLC cells *in vitro*, the role of circAGFG1 in the NSCLC tumor growth was also studied *in vivo*. circAGFG1 silencing notably reduced the NSCLC tumor growth *in vivo*.

Overall, high expression of circAGFG1 and HIF-1α might be a potential marker for the dismal prognosis of NSCLC patients. circAGFG1 promoted the malignant behaviors of NSCLC cells through accelerating glycolysis via targeting the miR-28-5p/HIF-1α axis.
